# Evolving Synaptic Plasticity with an Evolutionary Cellular Development Model

**DOI:** 10.1371/journal.pone.0003697

**Published:** 2008-11-11

**Authors:** Uri Yerushalmi, Mina Teicher

**Affiliations:** 1 The Leslie and Susan Gonda Interdisciplinary Brain Research Center, Bar-Ilan University, Ramat-Gan, Israel; 2 The Leslie and Susan Gonda Interdisciplinary Brain Research, Center, Bar-Ilan University, Ramat-Gan, Israel; Indiana University, United States of America

## Abstract

Since synaptic plasticity is regarded as a potential mechanism for memory formation and learning, there is growing interest in the study of its underlying mechanisms. Recently several evolutionary models of cellular development have been presented, but none have been shown to be able to evolve a range of biological synaptic plasticity regimes. In this paper we present a biologically plausible evolutionary cellular development model and test its ability to evolve different biological synaptic plasticity regimes. The core of the model is a genomic and proteomic regulation network which controls cells and their neurites in a 2D environment. The model has previously been shown to successfully evolve behaving organisms, enable gene related phenomena, and produce biological neural mechanisms such as temporal representations. Several experiments are described in which the model evolves different synaptic plasticity regimes using a direct fitness function. Other experiments examine the ability of the model to evolve simple plasticity regimes in a task -based fitness function environment. These results suggest that such evolutionary cellular development models have the potential to be used as a research tool for investigating the evolutionary aspects of synaptic plasticity and at the same time can serve as the basis for novel artificial computational systems.

## Introduction

Much recent effort has been directed towards understanding the mechanisms that underlie neural system development and plasticity by simulating biological processes at a time- scale of individual development. Intensive attempts are also under way to develop models that evolve artificial neural networks. However, most models evolving neural systems either lack the flexibility to handle a wide range of biological phenomena or do not have the requisite foundation to be considered biologically plausible.

The most common encoding used to evolve neural networks from genotypes is known as “direct encoding” where the phenotype information is directly encoded in the genome [1]. Other methods include parameterized encoding [2]–[4], which is usually restricted to predefined architectures, and non- modularity and grammar encoding [5]–[7], where the genome encodes a set of grammar rules that are used to build the phenotype.

Recently, several artificial evolutionary systems that incorporate a developmental phase have been presented. Various names have been suggested for such systems, including Artificial Ontogeny [8], Computational Embryogeny [9], Cellular Encoding [10], [11], Morphogenesis [12], and Artificial Embryogeny [13]. Some of these computational models enable evolving neural networks by simulating metabolic and cellular development processes [14]–[17].

In [18], an artificial genome entirely based on template matching in a nucleotide-like sequence was presented and used to study the dynamics of gene expression. In this model, as in the model presented in this paper, genes activate or inhibit other genes by producing products that bind to their regulatory sequences. However, this model has not been used for examining phenomena on a larger scale, such as at the tissue or neural level.

In [19], a simulation of complex biologically- inspired development was shown to be possible by evolving an organism through many cycles of cell division, differentiation and axonal growth. However, hand- written genetic instructions were used to control the organism's development.

In [10], grammatical rules were used to build the phenotype, but unlike grammar encoding [5]–[7], the grammatical rules were applied to a neural cell rather than a matrix. Each cell had a copy of the genome, which directly encoded a grammar tree. Each cell reads the grammar tree at a different position. Depending on what the cell reads, it can divide, change its internal parameters, and take on its final form as a neuron.

A similar grammar-based model for generating a neural phenotype was presented in [20]. The development starts with a germ cell that is represented by the start symbol of the grammar. However, these grammar based and cellular based encodings lack the ability to deal with complex interactions among the different developmental phenomena.

A model of neural network biological development based on a regulatory genome was presented in [21]. The NN was evolved as the controller of a virtual foraging organism.. In this model, an organism's NN development starts with a single cell that has 23 ‘proteins’. Some of these are extra-cellular signaling receptors, whereas others are responsible for the execution of developmental events, and still others are regulatory elements for the modulation of gene expression.

In [19], organisms evolve through many cycles of cell division, differentiation, and axonal growth. This study shows that a simulation of complex biologically inspired development is possible and can be successful. However, many of the genetic instructions to control the organism's development were hand-written.

A computational model of neurogenesis based on metabolitic processes was tested in [17]. The model could evolve large neural networks. Another system for evolving 3D organisms using gene expression mechanisms was presented in [16].

However, the drawback of all the encodings presented above is that they are either not based on biological processes, or lack the multiple interactions among different developmental phenomena that result in the emergence of biologically plausible models. Thus, despite promising progress, none of these models has succeeded in replicating a wide range of biological phenomena, including ones that demonstrate synaptic plasticity.

It has been argued elsewhere [22], that success in finding an efficient indirect biological encoding model should provide us with simulation tools that can teach us a great deal about the organization and functioning of biological systems.

Synaptic plasticity has long been regarded as a potential mechanism for memory formation and learning. The most famous synaptic plasticity principle is known as Hebbian learning [23], where modifications in synaptic transmission efficacy are driven by correlations in the firing activity of presynaptic and postsynaptic neurons.

Over the last 30 years, a large body of experimental results on synaptic plasticity has been accumulated. The long- lasting enhancement of synaptic transmission, Long Term Potentiation (LTP) first reported in 1973 [24], along with its counterpart Long-Term Depression (LTD), has been the focus of an enormous amount of investigation. Several experiments on various neuronal systems have found that synaptic plasticity may also depend on accurate spike timing; Spike Timing Dependent Plasticity (STDP) [25]–[29].

The field of evolutionary robotics provides interesting approaches to evolving synaptic plasticity. In [30] a model that genetically encodes rules of synaptic plasticity with rules of neural morphogenesis was shown to be feasible.

In [31] an evolutionary model combines an integrate and fire neuron with a correlation-based synaptic plasticity model and developmental encoding. The results on a simple robot navigation task indicate that such a system may allow for the efficient evolution of large networks.

However, these models are usually not designed to be based on biological specifics either at the gene to protein transcription functionality level or at the level of neural mechanisms and are usually restricted to correlation based plasticity regimes alone. To this day no evolutionary biological model has been shown to have the ability to evolve different biological synaptic plasticity rules.

In our previous papers, we presented an indirect encoding framework [32] capable of evolving behaving organisms with regulated mitosis and differentiated cells. We showed that the model is capable of evolving behaviors based on neural control [32], [33]. The same model has been shown to evolve gene- related phenomena such as functional gene clustering [34], and produce biological neural mechanisms such as temporal representations [33].

In this paper we examine the model's ability to evolve simple virtual organisms with different biological synaptic plasticity rules, and show that the model can evolve various plasticity regimes observed in nature.

## Results

In the next sections we present an evolutionary simulation model. The first section describes the chromosome model that is based on DNA and protein-like sequences. Two such chromosomes can reproduce an offspring chromosome, as detailed in the materials and methods section. The translation model for chromosomes to gene-protein networks and the gene-network dynamic system model is detailed later, preceding sections describing the way the cellular dynamics are translated into organism and cellular functionality. After presenting the model we report the results of several experiments. The first experiments use fitness functions that were directly designed to develop various synaptic plasticity regimes. The final, more complex, experiment uses a fitness function based on behavior.

More information about the framework, together with biological rationale for the model, can be found in [32]–[34].

### Chromosome

Each organism in the model expresses a phenotype derived from a chromosome structured according to biological conventions. Each chromosome includes a sequence of genes, in which each gene starts with a promoter sequence followed by a messenger RNA sequence.

Each promoter sequence has 1–3 cis-regulatory elements which are used as binding sites that regulate the expression of the gene, and a parameter block that includes the gene parameters. The parameter block of a gene/protein represents the properties derived specifically from its spatial structure. The use of gene and protein parameters in building the network is detailed later. Table 1 presents a list of all the parameters. Each mRNA sequence starts with a cis-regulatory element which is used as binding site that regulates the activation of the protein, followed by a parameter sequence, which in turn is followed by a trans-acting element which binds to other cis-regulatory elements to control their expression and activation; all represent the translated protein. All the cis-regulatory elements, trans-acting elements and parameter sequences are represented as sequences of real numbers, with the chromosome composed of a long sequence of real numbers *r_1…_r_n_*. The chromosome is translated into a gene-protein network as detailed in the following sections.

**Table 1:** Molecule parameters used in the gene-protein network dynamics.

Symbol	Description	Value Origin
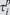	Production time constant for molecule *i*.	P_1_
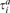	Activation time constant for molecule *i*.	P_2_
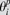	Production threshold for molecule *i*.	P_3_
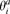	Activation threshold for molecule *i*.	P_4_
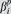	Production slope for molecule *i*.	P_5_
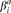	Activation slope for molecule *i*.	P_6_
*k_i_*	Diffusion coefficient of molecule *i*.	P_7_
*α_j_*	Intrinsic activity level of molecule *i*.	P_8_
**b**	A vector of 2 Boolean values indicating whether molecule *j* produces proteins inside the cell or whether it is bound to the membrane and affects external concentrations and whether molecule *i* is activated from the internal or external side of the membrane.	P_9_,P_10_
***k_type_***	A vector of Boolean parameters that governs the translated protein's ability to diffuse between soma-axon, soma-dendrite, synapsed dendrite-axon.	P11,P12
*w_ij_*	Connection strength between molecules *i* and *j*.	Hamming distance between rounded off values of the *〈trans〉* element of *j* and the *〈cis〉* element of *i*.

Each parameter value is derived from the chromosome, either directly through the parameter element, as most of the table entries, or using a Hamming distance function, as in wij. The parameters above are encoded for each gene/protein in the chromosome as a “parameter block” and govern the gene and its derived protein dynamics in the gene-protein network. The model separates the activation dynamics, controlling the ability of the gene-protein to affect other gene-proteins, and the production dynamics that control the protein's concentration, by having different slopes *β* ^•^, thresholds *θ* ^•^, and time constants *τ* ^•^ : *β^a^*, *θ^a^*, *τ^a^* for each gene/protein to control the dynamics of the activation and *θ^p^*, *β^p^*, *τ^p^* to control the dynamics of the protein production.

All experiments mentioned in this paper are based on simulations of evolutionary processes using genetic algorithm manipulations of such chromosomes. Mutation and crossover methods are discussed in the Reproduction subsection in Materials and Methods.

The chromosome structure described above can also be represented as a formal artificial chemistry grammar [35]–[37] in which the derivation rules are as follows:
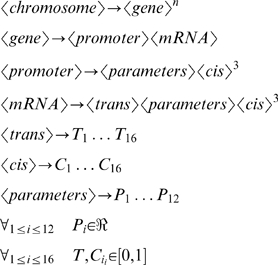



The language *L(〈gene〉)* created from the grammar based on the derivation rules above and *〈gene〉* as the start variable represents all possible genes in the chemistry. The language *L(〈mRNA〉)* is based on the same grammar, but with *〈mRNA〉* as a start variable and represents all possible proteins in the chemistry.

A molecule in the chemistry can either be a protein or a gene. Consequently, the set of possible molecules S can be written as:
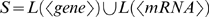



The model assumes that the set of molecules in an organism or cell is composed solely of the individual's genes and transcripted proteins.

### Gene-Protein network

**Figure 1:**
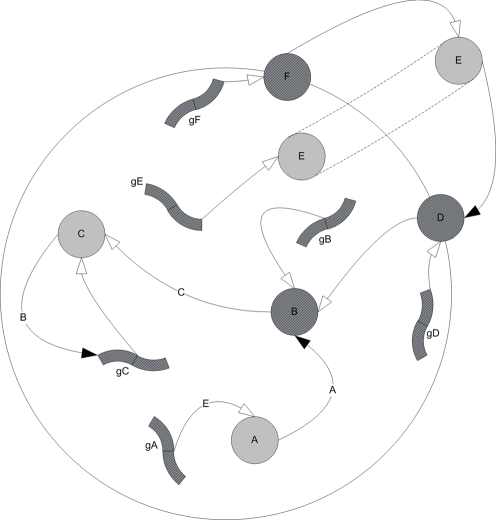
Example of a schematic simple-regulation network derived from a 6-gene chromosome. gA–gF are genes and A–F are their corresponding transcripted proteins. Proteins B, D & F act as productive proteins of C, B & E respectively. The white arrows represent production connections whereas the black arrows represent activation connections. Protein E can be produced inside the cell by its gene gE, and out of the cell by protein F, generating a cascading information system where E plays the role of a ligand, D is its receptor, and B and C are the internal messengers that eventually activate gene gC.

The chromosome presented above is translated into a gene-protein network as illustrated in Figure 1. The network connection strengths *w_ij_* are assigned according to the Hamming distance *d_ij_* between cis-regulatory elements and trans-acting elements. Each gene and each transcribed protein has 12 parameters (P_1_…P_12_) that are read from the chromosome and control its dynamics as detailed in Table 1.

The gene-protein network controls three dynamic values for each protein *i*:


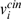
 - Protein *i*'s concentration inside the cell. 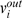 - Protein *i*'s concentration outside the cell, and 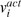 - the activity level of protein *i* in the cell. This value represents the extent to which the current spatial structure of the protein enables it to act on other genes and proteins.

The dynamics of the system are based on the following reaction rules:
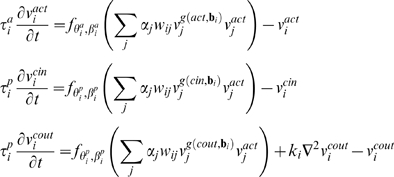
where 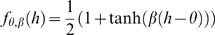,

The equations above are based on a threshold logic paradigm commonly used in simulations of genetic regulatory circuits [38], [39], and neural networks [40], [41], where the basic differential equation is of the form:
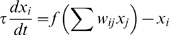



In such an equation the dynamics of a node value *x* are controlled by a time constant *τ*, and an activation function *f* that processes the cumulative field induced by the other nodes.

In the model, the field induced by a node *j* on node *i* is the product its dynamic activity level 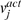, its concentration 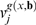, its static activity factor *α_j_*, and the connection between the nodes *w_ij_*.

To enable the model to separate the activation dynamics and the production dynamics, for example to affect a protein's concentration without affecting its spatial structure and vice versa, each gene/protein possesses different slopes *β* ^•^, thresholds *θ* ^•^, and time constants *τ* ^•^: *β^a^*, *θ^a^*, *τ^a^* to control the dynamics of activation and *θ^p^*, *β^p^*, *τ^p^* to control the dynamics of protein production.

The model enables the external concentration 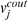 of each protein to play a role in the network dynamics by incorporating the expression 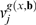 in the equations above. 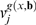 is either the internal 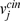 or external concentration 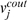, according to the values of x and b, which makes the model capable of evolving receptor-ligand relationships, based on the Boolean parameter b.

In order to permit tissue related dynamics, the external concentration equation contains a diffusion expression. *k_i_* is the diffusion coefficient of *i*, and 
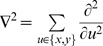
, so that the expression 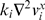 represents the contribution of diffusion to the change in external concentration, according to the diffusion equation 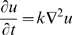.

An example of a schematic simple-regulation network derived from a small chromosome is shown in Figure 1.

### Cell functionality

In order to enable the gene-protein network presented above to model processes at the tissue level, output nodes were added to the gene-protein network. A similar component was introduced in [39] as a grammar of rules which describe inter- cell interactions and changes in number, type and state of cells. In our model, there is an output node *m* representing (i) a cellular- related event that can be triggered by the network (such as apoptosis, mitosis, cellular migration), or (ii) values that need to be derived from the network (e.g. Na conductivity, synaptic weight regulation), including modeling directional receptors for axon guidance, or (iii) values that need to be derived from the genome (such as translocation probability). The full list is shown in Table 2.

**Table 2:** List of all functions used in the direct fitness function experiments.

Description	Symbol	Output Type	Predefined Range
Mitosis messenger		B	{T,F}
Apoptosis messenger		B	{T,F}
Migration speed soma		A	(0,0.1)
Migration speed neurite		A	(0,0.1)
Sprout axon messenger		B	{T,F}
Sprout dendrite messenger		B	{T,F}
Translocation Probability		A	(0,1)
Soma Migration Directional Marker		C	(0,*2π*)
Axon Migration Directional Marker		C	(0,*2π*)
Dendrite Migration Directional Marker		C	(0,*2π*)
Crossover Probability		A	(0,1)
Axon Target Select Marker		B	{T,F}
Synapse Weight Axon	*ω_axon_*	A	(0,1)
Synapse Weight Dendrite	*ω_dendrite_*	A	(0,1)
Inhibitory Neuron Marker		A	(0,1)
Threshold potential	*θ*_0_	A	(-60E-3,-70E-3)
Threshold adaptivity factor	*α*	A	(0.005, 0.05)
Threshold time constant	*τ_θ_*	A	(15E-3,50E-3)
gNa in open channel state		A	(4.0,4.4)
gNa in closed channel state		A	(20E-3,50E-3)
Action Potential Refractory Time	*τ_ref_*	A	(2E-3,5E-3)
k Refractory Time	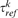	A	(4E-3,6E-3)
gk in open channel state		A	(200E-3, 500E-3)
gk in closed channel state		A	(2.3,2.6)
Synaptic current rise time	*τ_s_*_1_	A	(0.5E-3,2E-3)
Synaptic current decay time	*τ_s_*_2_	A	(3E-3,7E-3)
Membrane time constant at rest:	*C*/Σ*_g_*	A	(0.005, 0.02)
Neural noise time constant		A	(0, 1E-2)

The function values were limited to be in the ranges above. Type ‘A’ functions transform u_m_ linearly to be in a predefined (min,max) range. Type ‘B’ functions are Boolean functions based on a u_m_>0 test. Output Type ‘C’ functions are directional functions and are based on 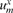, 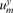 & umy detailed earlier and produce an angle. All predefined ranges were chosen to cover reasonable biological values. Migration speed values are given in cell diameters per epoch. Neural electric properties are given in OASM (Ohm, Ampere, Second, Meter) like units, in the simulations each epoch represented half a millisecond. Only symbols of values that are referred to by symbol in the text are presented.

Each such output node *m* is represented by a random-generated bit string *s_m_*. The protein nodes *j* in the gene-protein network that are close enough to string *s_m_* (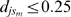) are connected to output node *m*. According to the threshold logic paradigm, an internal value *u_m_* is calculated for each output node:
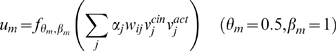



For nodes that trigger an event (e.g., occurrence of mitosis, cell death, migration, differentiation event), the event is triggered when the value *u_m_* exceeds a predefined threshold (0.5). When managing scalar values such as a translocation probability, the internal value *u_m_* may be multiplied by another pre-defined factor to obtain the actual scalar value as detailed in Table 2.

In cases where a receptor-ligand relationship was needed to obtain directional quantification (the ability to choose a direction, as in axon guidance or cell migration), a two dimensional version of the above value was used, where the effect of internal factors was replaced by the effect of external gradient factors:
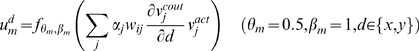



A list of all functions used in this paper is detailed in Tables 2 and 3. As seen in all tables, all parameters were allowed to evolve within a biologically reasonable range, and no parameter was pinned to a specific value.

**Table 3:** List of additional functions used in the behavior based fitness function experiments.

Description	Symbol	Output Type	Predefined Range
Cell differentiation messenger		B	{T,F}
Sensory neuron marker		A	(0,1)
Motor neuron marker		A	(0,1)
Hidden neuron marker		A	(0,1)
Odor A Sensor Marker		A	(0,1)
Odor B Sensor Marker		A	(0,1)
Sight Sensor Marker		A	(0,1)
Photoreceptor sight angle	*α_pr_*	A	(0, *π/2*)

These functions were added to the ones detailed in Table 2 in the behavior- based experiments. The function values were limited to be in the ranges above. Type ‘A’ functions transform u_m_ linearly to be in a predefined (min,max) range. Type ‘B’ functions are Boolean functions based on a u_m_>0 test. All predefined ranges were chosen to cover reasonable biological values. Only symbols of values that are referred to by symbol in the text are presented.

In this paper the term ‘organism’ refers to the group of all cells that are repeated-mitosis results of the same zygote cell. Since during mitosis the gene-protein network is copied from the parent cell, all organism cells are controlled by the same network structure, but since each cell is situated in a different location, it may possess different internal and external protein concentrations.

**Figure 2:**
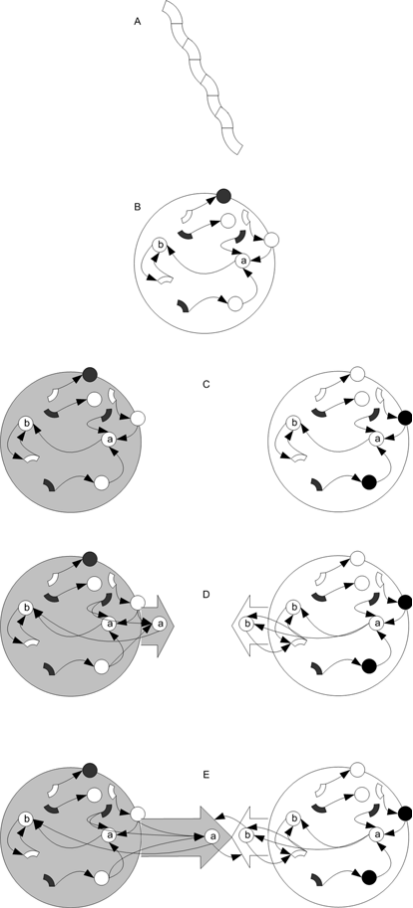
Synapsogenesis scheme example. A) The basic building plan for the cellular tissue is the chromosome. B) The chromosome is translated into a zygote controlled by a regulatory network. C) A mitosis event splits the zygote into two separate cells, where each cell has its own instance of the same regulatory network template. D) Neurite sprouting events occur in both cells. An axon is branched from the left cell, and a dendrite – from the right cell. Proteins a and b are marked in P11 as ones that cannot diffuse from neurite to soma. Therefore, their instances are separated into neurites with the same connectivity. E) After the axon is guided by external protein concentrations towards the right cell's dendrite, a ‘synapsogenesis’ event occurs. A synapse is formed, allowing proteins marked as synapse-diffusible (in their ktype parameter) to move from one cell to another.

To control dendrites and axons separately from their soma, we needed a mechanism to separate the dynamic values of the cell's soma from those of a neurite. Therefore, another basic parameter (P_11_) was added to control the protein's ability to diffuse between a cell's soma and neurite. Based on this diffusion parameter, the system decides whether a given protein in a neurite will have the same dynamic properties as the soma's and be presented by the same node in the regulatory network, or whether it will have separate properties based on a different node connected to the same regulatory network. A simple example of synapsogenesis is illustrated in Figure 2. In the experiments, the weight value of a synapse connecting axon A to dendrite B is the sum of the axon's and dendrite's synapse weight functions *ω_axon_* and *ω_dendrite_* as indicated in Table 2:

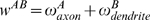



### Organism development

Each organism is initially made up of a single cell that has no initial external or internal concentrations. The initial cell's genes can produce proteins that change its internal and external concentrations and may cause some functional events such as mitosis or cell migration. The cells can repeat mitosis events, so eventually the organism can consist of several cells, where each cell is controlled by the same chromosome and has the same controlling regulatory network, but may have different internal and external concentrations. The internal and external concentrations are changed dynamically by the regulation network and diffusion rules presented above. The cells are represented as circles on a two- dimensional grid and can move to continuous position values. In a cell division event, the daughter cell is adjacently located on an axis according to the “Soma Migration Directional Marker” (see Table 2) of the mother cell. When a “Sprout axon/dendrite Messenger” is triggered, a neurite is sprouted from the soma. All cell elements (soma, axon and dendrite) can migrate on the grid according to their migration speed and directional marker (see Table 2). When an “Axon Target Select Marker” event is triggered in an axon, and the axon has a dendrite at a distance of less than one cell radius, the axon synapses to the nearby dendrites that have the same flag are turned on.

**Figure 3:**
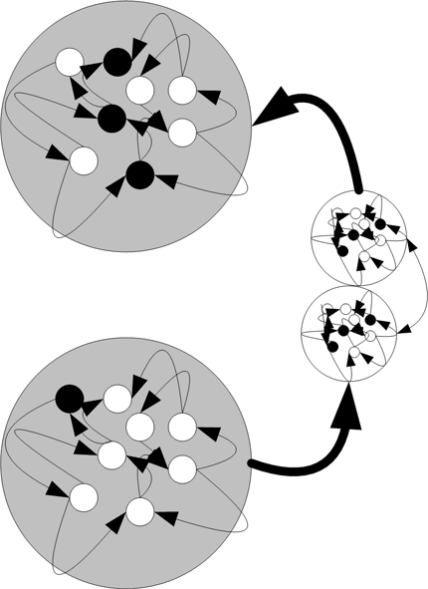
Outcome of the first period in the direct fitness function experiments. The outcome of the first period in the direct fitness function experiments is a dual cell organism in which a synapse was formed between an axon of one cell and the dendrite of the other. The grey circles represent the somas of two cells. The smaller white circles represent axons and dendrites. The internal networks represent the gene-protein networks in each soma, axon or dendrite that all have the same connections, but may have different states.

**Figure 4:**
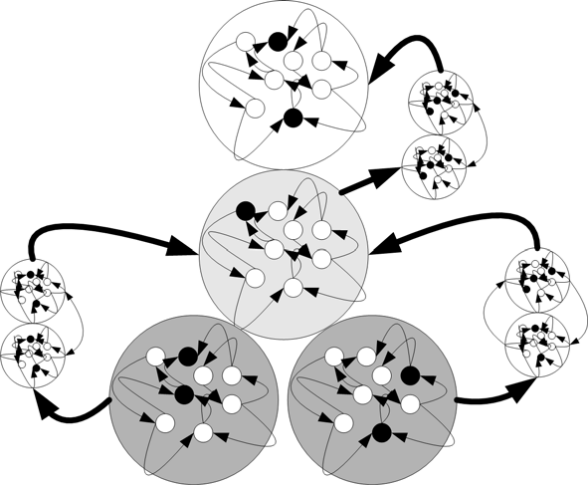
An example of a simple organism developed in the behavior- based experiment. A simple organism developed in the behavior- based experiment. This specific organism has two (bottom) motor cells, which synapse to a hidden cell (middle), which synapse to a sensor cell (top). The large circles represent somas of cells, the smaller white circles represent axons and dendrites. The internal networks represent the gene-protein networks in each soma, axon or dendrite that all have the same connections, but may have different states.

The organism has a certain period of time in which it has to stop mitosis; only then will the organism be an adult that may reproduce. If the organism does not stop mitosis during the predefined period, it is regarded as a cancerous tissue and removed from the environment without reproduction. Figure 3 illustrates an organism developed in the direct fitness function experiments presented in this paper. An example of an organism that can develop in the behavior- based experiments is illustrated in Figure 4.

### Direct Fitness Function Experiments

In these experiments, the ability of the model to evolve different synaptic plasticity regimes was tested on a two- dimensional 10×10 cellular grid. The physical environment included action molecules *m* as detailed in Table 2. All guidance actions were based on chemical attraction in the extra-cellular environment.

**Figure 5:**
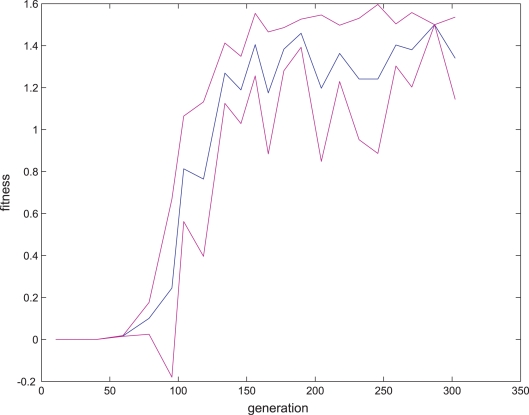
The first evolution period in the direct fitness function experiments. The process of evolving organisms in the first period in the direct fitness function experiments. Twenty eight generations were randomly selected through evolution. Blue line indicates the average fitness in each generation; purple lines indicate error range of one standard deviation.

Forty-five evolutionary sessions were run, with a fixed population size of 100 and simple roulette wheel selection [42], where the probability *p_i_* of an organism *i* to be selected is associated to its fitness *f_i_* according to:
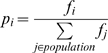



A two- period fitness function was defined:

- First period, evolving organisms with only two neurons and a single synapse between them:
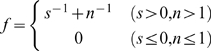
where *f* is the fitness value, *s* is the number of synapses between two different cells, and *n* is number of neurons. The optimal organism structure in this fitness landscape is an organism with two neurons connected by a single synapse. This period was run until the entire population reached maximal fitness. A sample fitness curve for a first period session is presented in Figure 5.
- Second period, evolving the first period organisms to achieve target synaptic plasticity properties.

As will be detailed later, the fitness function in the second period was changed every 8 evolutionary sessions and included a regression calculation (see regression calculation section in Materials and Methods) of samples based on:

- Δ*w_t_* - Synaptic change at time *t* compared to the next system epoch. Δ*w_t_* = *w_t+1_-w_t_*.

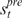
 - Time interval from last pre-synaptic cell spike up to time *t*.

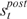
 - Time interval between last post-synaptic cell spike up to time *t*.

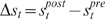
. The interval from the last pre- and post -synaptic cell spikes at time *t*.

Organisms that did not have exactly two connected neurons were not allowed to reproduce in this period.

### Evolving Hebbiann LTP and Anti-Hebbiann LTD Synapses

**Figure 6:**
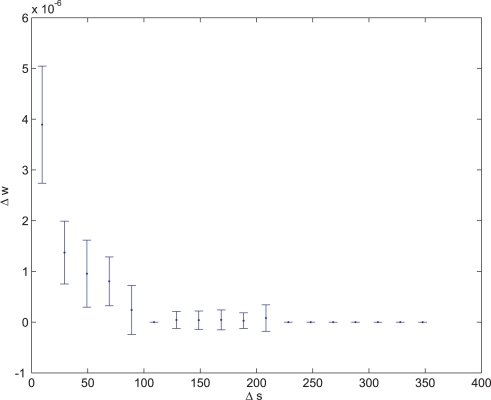
Average synaptic change plotted against spike time measures in Hebbian- like LTP fitness sessions. Hebbian- like LTP plasticity; synaptic change plotted against Δ*s_t_* (defined as the interval between the pre-synaptic spike and the post-synaptic spike), taken from a 30th generation virtual organism evolved in a session with fitness function 
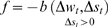
. Spike time measures are given in system epochs, synaptic change Δ*w_t_* is given in absolute synaptic weight values. Each plot presents the average synaptic change values from 24 sessions of 1000 epochs. The error bars are set at one standard deviation.

The Hebbian postulate assumes an increase in synaptic strength when the pre-synaptic cell “takes part” in the firing of the post-synaptic cell [23]. Assuming that times *t* with small values of Δ*s_t_* are characterized by spikes of the post-synaptic cell affected by the pre-synaptic cell, we examined the model's ability to evolve synapses when the synaptic change Δ*w_t_* is dependent on the interval between the pre- and post- synaptic spikes. We defined the following fitness function to evolve Hebbian LTP synapses:
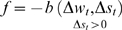
where 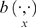 is the regression coefficient of samples that meet condition *x*. An example of such a synapse is presented in Figure 6.

**Figure 7:**
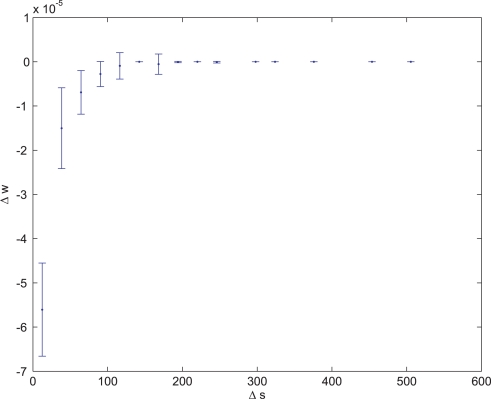
Average synaptic change plotted against spike time measures in the Anti-Hebbian-like LTD fitness sessions. Anti-Hebbian-like LTD plasticity; synaptic change plotted against Δ*s_t_*, taken from a 30th generation virtual organism evolved in a session with fitness function 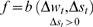. Spike time measures are given in system epochs; synaptic change Δ*w_t_* is given in absolute synaptic weight values. Each plot presents the average synaptic change values from 24 sessions of 1000 epochs. The error bars are set at one standard deviation.

**Figure 8:**
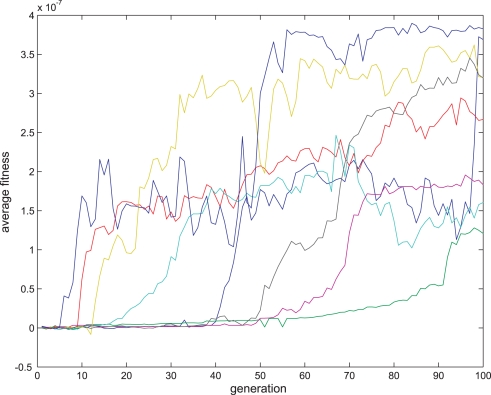
Fitness curve of evolving Hebbian LTP synapses using the direct fitness function. Average fitness values in every generation for the eight sessions running the Hebbian- like LTP fitness function. The first generation is the outcome of an evolutionary session designed to evolve organisms with only two neurons and a single synapse between them as detailed in the text.

**Figure 9:**
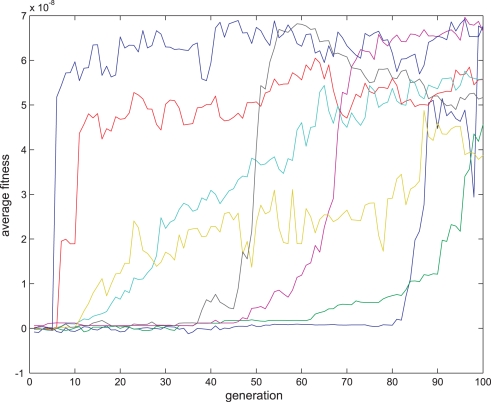
Fitness curve of evolving Anti-Hebbian LTD synapses using the direct fitness function. Average fitness values in every generation for the 8 sessions running the Hebbian- like LTD fitness function. The first generation is the outcome of an evolutionary session designed to evolve organisms with only two neurons and a single synapse between them as detailed in the text.

We also assumed that the opposite function may generate Anti-Hebbian LTD synapses:
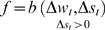
An example of such a synapse is presented in Figure 7. Eight sessions were run in each evolutionary session. The fitness curves are presented in Figures 8 and 9. Statistical tests of all populations at the 100^th^ generation showed that in all sessions the proportion of organisms where the regression was significantly (P<0.05) negative (LTP) or positive (LTD) was above 81%. In the above sessions we ignored samples in which Δ*s_t_*≤0.

### Evolving Non-Hebbian LTP Synapses

After the model successfully evolved synaptic plasticity based on pre-to-post synaptic correlations, we examined the ability of the model to evolve synaptic plasticity that was not connected to such a correlation by defining a fitness function that prefers a dependence of synaptic change on the interval since last pre- or post- synaptic spike.

**Figure 10:**
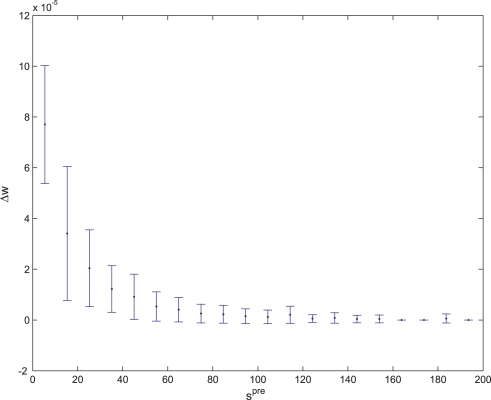
Average synaptic change plotted against spike time measures in Non-Hebbian LTP pre-synaptic plasticity fitness sessions. Non-Hebbian LTP pre-synaptic plasticity; synaptic change plotted against 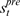 (defined as the time interval since the last pre-synaptic spike), taken from a 20th generation virtual organism evolved in a session with fitness function 
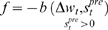
. The post-synaptic cell was set as non-active during measurements. Spike time measures are given in system epochs; synaptic change Δ*w_t_* is given in absolute synaptic weight values. Each plot presents the average synaptic change values from 24 sessions of 1000 epochs. The error bars are of one standard deviation.

**Figure 11:**
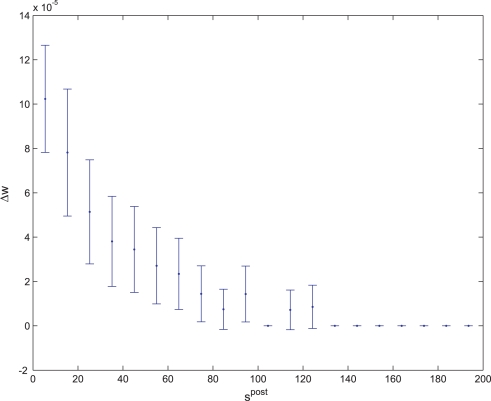
Average synaptic change plotted against spike time measures in Non-Hebbian LTP post synaptic plasticity fitness sessions. Non-Hebbian LTP post-synaptic plasticity; synaptic change plotted against 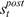 (defined as the time interval since the last post-synaptic spike), taken from a 20th generation virtual organism evolved in a session with fitness function 
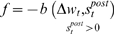
. The pre-synaptic cell was set as non -active during measurements. Spike time measures are given in system epochs; synaptic change Δ*w_t_* is given in absolute synaptic weight values. Each plot presents the average synaptic change values from 24 sessions of 1000 epochs. The error bars are set atone standard deviation.

**Figure 12:**
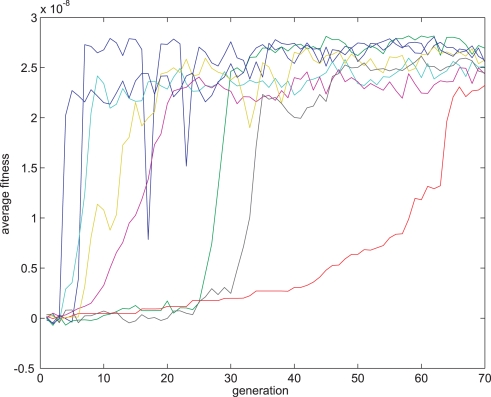
Fitness curve of evolving Non-Hebbian LTP pre- synaptic synapses using the direct fitness function. Average fitness values in every generation for the eight sessions running the Non-Hebbian pre-synaptic LTP fitness function. The first generation is the outcome of an evolutionary session designed to evolve organisms with only two neurons and a single synapse between them as detailed in the text.

**Figure 13:**
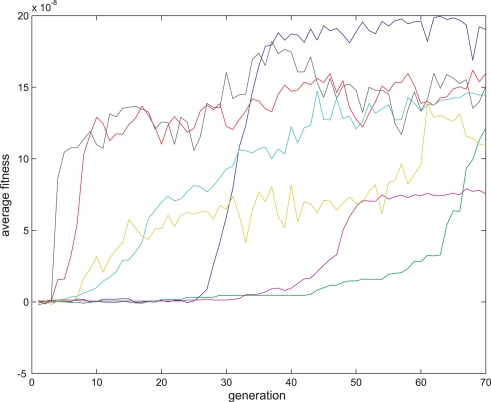
Fitness curve of evolving Non-Hebbian LTP pre synaptic synapses using the direct fitness function. Average fitness values in every generation for the eight sessions running the Non-Hebbian post-synaptic LTP fitness function. The first generation is the outcome of an evolutionary session designed to evolve organisms with only two neurons and a single synapse between them as detailed in the text.

The following fitness functions were set to evolve synapses that strengthen upon single neural activity of the pre-synaptic cell:
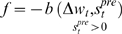
or the post-synaptic cell:
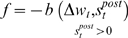
In these experiments the fitness function was based on the regression coefficient of the synaptic change vs. the time interval since the last spike. To control that the synaptic change was not connected to a pre- or post- synaptic correlation, the measurements to test synaptic plasticity were made after stopping the supplementary cell's activity. Examples of evolved Non-Hebbian pre- and post- synaptic LTP synapses are shown in Figures 10 and 11 respectively. The fitness curves are presented in Figures 12 and 13. Statistical tests of all populations in the 70^th^ generation showed that in all sessions the proportion of organisms where the regression was significantly (P<0.05) negative was above 93%.

### Evolving Time Dependent Synaptic Plasticity

**Figure 14:**
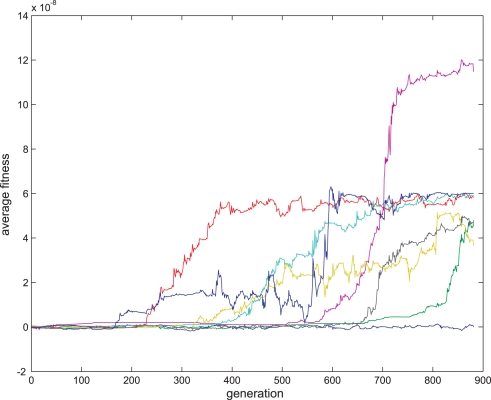
Fitness curve of evolving STDP synapses. Average fitness values in every generation for the eight sessions running the STDP fitness function. The first generation is the outcome of an evolutionary session designed to evolve organisms with only two neurons and a single synapse between them as detailed in the text.

**Figure 15:**
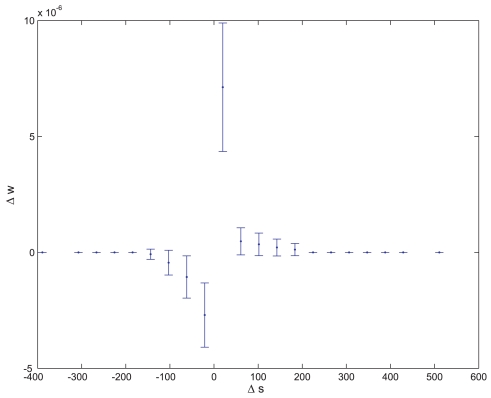
Hebbian- like STDP. Synaptic change plotted against Δ*s_t_*, taken from a 400^th^ generation virtual organism evolved in a session with fitness function:
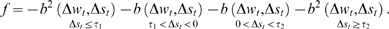
Spike time measures are given in system epochs; synaptic change Δ*w_t_* is given in absolute synaptic weight values. Each plot presents the average synaptic change values from 24 sessions of 1000 epochs. The error bars are set at one standard deviation.

Finally, eight sessions were run in an attempt to evolve a more complicated form of synaptic plasticity which was time-dependent (STDP). By contrast to the previous plasticity regimes which evolved in fewer than 100 generations, we did not obtain such a clear plasticity rule in the STDP case using the following fitness function:
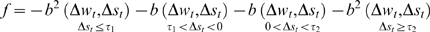
As seen in Figure 14, after 881 generations, seven out of eight evolutionary sessions produced a satisfactory plasticity regime. Statistical tests on seven out of the eight populations at the 881^th^ generation showed that in these sessions the proportion of organisms where the synaptic change was significantly (P<0.05) positive or negative when the interval between the pre-synaptic and post- synaptic spikes was small and positive or negative respectively and where the regression was only significantly (P<0.05) negative in small intervals was above 83%. The STDP of one of the evolved synapses is shown in Figure 15, and is comparable to previous biological findings [29].

### ***k_type_*** as an essential model component

The *k_type_* parameter (see Table 1) that regulates the protein's ability to diffuse between axon-soma-dendrite is a relatively new element in our model. Since the previous fitness curves implied that the Non-Hebbian LTP synapses were relatively easy to evolve we examined whether this parameter was essential for evolving these synapses. We ran four evolutionary sessions of 100 generations each, where ***k_type_*** was not used in the model and all proteins were allowed to diffuse freely between soma-axon and soma-dendrite. In all four sessions, the population could not evolve a clear positive fitness value.

### Behavior Based Experiments

We next examined whether any of the plasticity regimes could evolve in an environment where the evolutionary pressure was based on the organism's behavior.

In this experiment, each virtual organism was randomly set to be a male or a female, and could move in the environment by using its sensor neurons (that cannot have dendrites), motor neurons (that cannot have axons) and hidden neurons (that can have both dendrites and neurons) as detailed later. In order to encourage the virtual organisms to develop neural networks, they were given a life span proportional to the different cellular types they developed: a sensory neuron, a motor neuron, a hidden neuron, a dendrite, an axon, and a synapse. Hence, a maximal time span was assigned to every virtual organism that possessed a “basic” neural network, which was defined as having at least one instance of each of the six elements mentioned above. Since the system was defined as having only dendrite-to-axon synapses, the “basic” network can also be seen as a network that included at least a motor, a hidden, and a sensory neuron and one synapse. The virtual organisms were removed from the environment after completing their life span period.

The population size was restricted to a predefined range by removing the eldest virtual organisms from the environment when the number of virtual organisms reached the upper bound (due to crowding), and by randomly choosing two parents from the environment and producing their offspring as a new individual in the environment when the number of virtual organisms reached the lower bound.

Each virtual organism in the behavioral based experiments was developed on a two-dimensional 20×20 cellular grid. The physical environment included action molecules *m* as detailed in Tables 2 and 3.

The lower and upper bounds of the population size were set to 90 and 110. Unlike previous evolutionary sessions, in this session a specific fitness function was not set and therefore a roulette wheel selection [42] was not used. The evolutionary pressure was set only by including the life-span period in the environment as a function of the neural network structure, and introducing a “mating rule” (see below). Unlike the previous experiments, in this experiment successive generations could overlap.

### Cell Differentiation

In order to incorporate behavior into the model, we needed to include the possibility for cell differentiation so that the organism could include sensor and motor cells in addition to its hidden neural cells. Briefly, when a cell differentiation messenger is triggered, the cell differentiates into one of three cell types according to its differentiation marker with the highest level (as detailed in Table 3):

- **A motor cell**. Upon firing, motor cells cause the virtual organism to move in the *l_m_-l_c_* direction, where *l_m_* is the motor cell location, and *l_c_* is the virtual organism's centroid.
- **A sensor cell**. Sensor cells are either sensitive to an odorant (A or B), or act as a photoreceptor. Odor A is emitted into the environment by potential mate organisms, odor B is emitted by non mate organisms; the secreted current *I* from an odor sensitive cell is proportional to the distance between the odorant origin and the cell. Photoreceptor cells secrete a constant current if any virtual organism is placed in a region subsumed by *α_pr_* radians.
- **A hidden cell** – that will embed a neural model as detailed in previous sections.

### The Evolutionary Session

In order to evolve neural based behavior, a “mating rule” was introduced in the environment, where two virtual organisms that contacted each other produced offspring according to the reproduction equation presented in the Materials and Methods section. Hence we expected the virtual organisms to develop neural mechanisms that would maximize their contacts with virtual organisms of the opposite sex.

**Figure 16:**
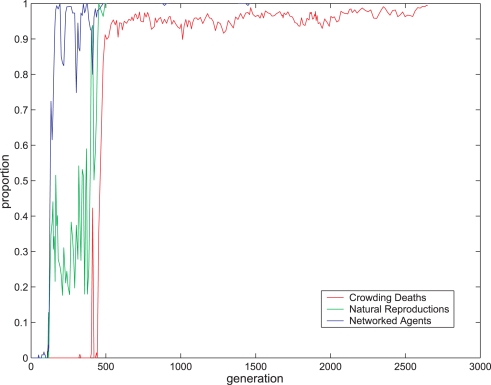
Evolution in behavioral experiment. Green: Proportion of reproduction triggered by virtual organisms contacting each other (as opposed to reproductions initiated by the system when the number of virtual organisms hit the lower bound). Blue: Proportion of virtual organisms that developed a basic network as defined in the text. Red: Proportion of virtual organism death events triggered by the system because of crowding (as opposed to deaths due to completing the life span period). The values are average proportions measured every 10 generations.

As a first step, we tested for changes in the virtual organisms' behavior along generations. As shown in Figure 16, the percentage of reproduction resulting from virtual organism contact rose over generations, which cannot be ascribed to a change in the population size (which, as detailed earlier, was always between 90 and 110).

In order to examine whether any of the synaptic plasticity regimes evolved in the behavioral experiment, every 0.5 generations (where the population generation was calculated as the average generation of the individuals in the population) an organism was randomly chosen from the population and tested to see whether it included (1) synapses, where the value of 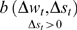 was negative at P<0.05. These synapses are termed Hebbian LTP synapses; (2) synapses where the value of 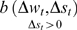 was positive at P<0.05, and are referred to as Anti-Hebbian LTD synapses, (3) synapses where the value of 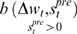 was negative at P<0.05, termed Hebbian pre-synaptic LTP synapses, (4) synapses where the value of 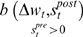 was negative at P<0.05, termed Non-Hebbian pre-synaptic LTP synapses. We did not control for STDP synapses in this experiment.

For each of the four synapse types above we examined whether there was an increase in the proportion of virtual organisms with the specific synapse type in the first 1000 generations compared to the second 1000 generation. There was an increase in the proportion of virtual organisms that had at least one Non-Hebbian pre-synaptic LTP synapse (P<9×10^−10^, T-test). No such findings were observed for the other synaptic plasticity types (P>0.1, T-test).

### Non-Hebbian pre-synaptic LTP synapse as a memory mechanism

This finding could be explained by (1) the tendency of the evolution to use Non-Hebbian pre-synaptic LTP synapses in this early stage for mating functionality or (2) some unrelated mechanism preferring such organisms in the latter period, such as a tendency of the organisms to include more synapses, which would increase their likelihood of including at least one instance of a specific synapse type.

**Figure 17:**
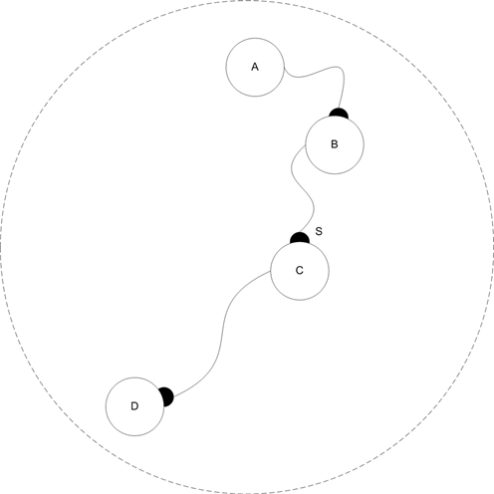
An organism with a simple memory mechanism of sensed potential mates. One of the developed organisms included a sensory neuron A that was sensitive to mate odors, synapsing a hidden neuron B, synapsing using a Non-Hebbian pre-synaptic LTP synapse S, a hidden neuron C, synapsing a motor neuron D. Neuron B firing at a high rate as a result of a proximal mate potentiates synapse S and immediately raises the firing rate of D , causing the organism to turn around and stay in the same area.

A manual examination of the virtual organisms that included a Non-Hebbian pre-synaptic LTP synapse gave us a clue to the use of these synapses. As illustrated in Figure 17, in some of the cases the Non-Hebbian pre-synaptic LTP synapse was in a path connecting asensors sensitive to a “mate” odor to a motor neuron. This caused the organism to turn around, resulting in a behavior where the synapse “remembered” that there had just been a potential mate nearby, causing the organism to remain in its vicinity and hence increasing the probability of encountering the previously sensed mate.

We tested the mutual information between synapse potentiation and the existence of a mate nearby. If the synapses function as a memory mechanism for “remembering” whether there are mates the virtual organism just sensed, we should observe higher mutual information values in cases of synapses recognized as Non-Hebbian pre-synaptic LTP synapses compared to randomly chosen synapses. We compared 250 virtual organisms with a Non-Hebbian pre-synaptic LTP synapse (group B) to 250 randomly chosen virtual organisms (group A). Each virtual organism was placed in an environment and we sampled 1000 instances of:

*d_t_*: the distance of the nearest mate at time *t*.
*w_t_*: the value of the Non-Hebbian pre-synaptic LTP synapse weight value at time *t*. For Group A organisms, a synapse was chosen that had some variance in the *w_t_* values (*Var(w_t_)>0*).

For each organism the *d_t_*, *w_t_* values were converted to Boolean values *D = {d^1^,d^2^}* and *W = {w^1^, w^2^}*:
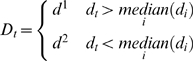


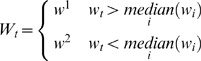



**Figure 18:**
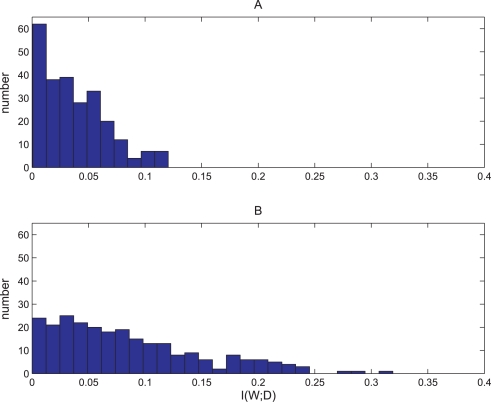
Histogram of mutual information values of virtual organisms with Non-Hebbian LTP synapses compared to randomly chosen synapses. A. Histogram of I(D;W) values of 250 randomly chosen synapses with var(W)>0 of different virtual organisms randomly chosen from the behavior- based evolutionary session. B. Histogram of I(D;W) values of 250 randomly chosen Non-Hebbian LTP pre- synaptic synapses of different virtual organisms randomly chosen from the behavior- based evolutionary session. I(D;W) is the mutual information between the synapse state and the distance of the closest mate. The Non-Hebbian LTP pre- synaptic synapses contained more information about the closest mate (T-test P<10^−16^). For more information about the way I(D;W) was calculated see the Materials and Methods sections.

As shown in Figure 18, there was a significant difference in mutual information *I(D;W)* between Group A and Group B.(T-test P<10^−16^). This suggests that the Non-Hebbian pre-synaptic LTP synapses tend to contain more information about the existence of potential mates in the immediate vicinity.

## Discussion

The experiments presented in this paper were designed to examine the ability of an evolutionary cellular development model to evolve various synaptic plasticity regimes. The approach described in this paper is based on a model that is both feasible in terms of running relatively complex evolutionary simulations and includes a biologically plausible gene-protein regulation functionality with an integrate-and-fire neural mechanism.

By applying different fitness functions to the model in separate evolutionary sessions, different synaptic plasticity rules could be evolved by the system: Hebbian- like LTP plasticity, Anti-Hebbian-like LTD plasticity, Non-Hebbian LTP pre- and post- synaptic plasticity and Hebbian- like STDP. We also showed how Non-Hebbian LTP pre- synaptic plasticity can evolve in an evolutionary system that has no direct fitness function, but is based on behavior selection, and how this plasticity can serve as a simple memory mechanism.

According to the fitness curves in the direct fitness function experiments, anti-Hebbian like plasticity regimes converge for most runs quite well, especially compared to the fitness curves of the other plasticity types, and therefore might have a selectional advantage in the last experiment.

Although in this paper we focused on the model's ability to evolve various synaptic plasticity regimes, we also examined the model as a whole and did not derive the specific features in the model that enable evolving synaptic plasticity. For example, even though the diffusion functionality does not seem to be related to the abilities of the model as presented in this paper, our attempts to evolve the simplest plasticity regime without diffusion did not succeed, suggesting that the ability to regulate protein diffusion between neurites and soma is essential. We believe that future studies should examine the ability of simplified models to evolve various synaptic plasticity regimes to discover new essential components of complex mechanisms.

The ability of the model to evolve various biological plasticity regimes, together with its ability to present genomic and neuronal phenomena suggest that this simulation approach can serve as a tool for investigating evolutionary aspects of synaptic plasticity. One example for such future examination is related to the connection between the time when STDP emerges on the biological phylogenetic tree and the number of generations needed to evolve it in the model. The fact that some synaptic plasticity rules such as time dependent plasticity evolved much more slowly than others as seen in the results presented here can be ascribed to unsuitable fitness functions.

A different conclusion relates to synaptic plasticity as a mechanism for memory formation and learning. The fact that the model is capable of evolving a biological synaptic plasticity rule as a pre- synaptic LTP synapse in an environment where selection is based on behavior and not directly designed to evolve this specific plasticity should encourage the computational applications of such an approach. We believe that future experiments using this and similar models can shed more light on other synaptic plasticity rules and their relationship to memory and learning, both for future artificial intelligence systems, and as a research tool to account for existing neural systems. Nevertheless, the fact that some synaptic plasticity rules, such as time dependent plasticity, evolved in the direct fitness experiments much more slowly than others, implies possible computation power difficulties in larger and more complex models. We believe that statistical examination of the artificial mechanisms that lead to the creation of more complex synaptic regimes in an evolutionary environment with behavior based selection has the potential to better understand synaptic plasticity both as a biological and computational research tool.

## Materials and Methods

### Reproduction

Reproduction of a child chromosome from its parent chromosomes is based on a self adaptive method [43], avoiding linkage of the experimental results to specific crossover and mutation values. Each real value *r_i_* (*P_i_*, *T_i_*, *C_i_* in the grammar above) of the chromosome is surrounded by three other values: a crossover probability value *c_i_*, and two mutability values 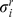, 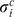 that control the extent to which parameters *r_i_* and *c_i_* respectively are likely to change (for more information see [43]). The values of *r_i_*, *c_i_*, 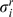, 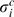 are mutated self-adaptively:
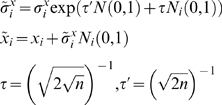
where *n* is the number of genes, *x*∈{*r*, *c*}, *i*∈{1‥*n*}, *N*(0, 1) is a standard normal random number, *N_i_*(0, 1) represents a new random number generated for each component, and 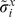, 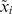 are the new values for 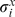, *x_i_*.

Before mutation takes place, the parent chromosomes are aligned using a dynamic programming algorithm [44] and recombined, where the probability for a crossover point to occur on the aligned chromosomes at location *i* & *j* of the parents is *c_i_+c_j_*.

### Neural activity

All cells in the experiments detailed in this paper were embedded with an integrate and fire [45] neural model, where the membrane potential of the cell body behaved according to:
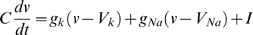
where *C* is the membrane capacitance, *I* is the total current injected into the cell, and *g* and *V* values are the ion channel conductivity and reversal potential.

When the membrane potential reaches threshold *θ*, and the cell is not refractory, it fires an action potential, *g_Na_*. It is then raised for one system epoch, and immediately switches to a refractory state for *τ_ref_* seconds, where it cannot fire and *g_k_* is raised and later decayed back with a 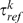 time constant. In the simulations each epoch represented half a millisecond.

Current *I* injected into the cell consists of a noise current *I_noise_*, and incoming synapse current *I_exc_*, where *I_noise_* is a Gaussian noise causing a cell without external input to fire randomly. The noise of the various cells is uncorrelated.
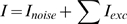



Excitatory *I_exc_* current injected by a pre-synaptic cell *i* into a postsynaptic cell *j* has a rise and decay time as follows:
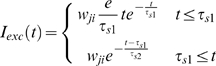
where *t* is the time elapsed from the last action potential in the pre-synaptic cell, and *τ*_1_ & *τ*_2_ are the rise and decay time constants.

The threshold level depends on the membrane potential level, according to:
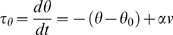



### Chromosome model

We used cis- and trans- elements as sequences of 16 real numbers. Since the connection strength between molecules *i* and *j*, *w_ij_* is calculated by the Hamming distance between rounded off values of the *trans* element of *j* and the *cis* element of *i*, the length of cis and trans elements controls the resolution in which *w_ij_* can be calculated.

Two evolutionary simulations were conducted to evolve Non-Hebbian pre-synaptic LTP synapses using 8 and 32 cis- and trans- lengths, and in both cases the sessions succeeded in evolving the synapses.

### Generating output nodes

Bitstrings *s_m_* representing the output nodes are set randomly during initialization of each evolutionary session.

### Calculating Regression

All regression coefficients and regression significance values presented in this paper were values derived from tests where the organism was placed in its environment and there were at least 2000 pre-synaptic and 2000 post-synaptic spikes from both sides of the synapse while sampling. No external current was injected into the neurons so as to collect spike related data. Synapses that did not have any pre-synaptic or post-synaptic spikes during the experiment were not included in the regression calculations.

### Mutual information

The mutual information *I(W;D)* was calculated according to:
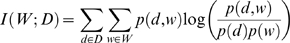
where *p*(*w*,*d*) is the number of samples of the joint *w*,*d* divided by the total number of joint samples. *p*(d) and *p*(w) are the number of samples of d and *w* respectively, divided by the total number of samples. The number of samples used was 1000. By letting the simulation progress between each two samples, two consecutive samples were not allowed to have the same closest “mate” to the examined organism.
